# Achieving sustainable development in China: a moderated mediation model of guanxi HRM practices

**DOI:** 10.3389/fpsyg.2025.1620530

**Published:** 2025-06-18

**Authors:** Xueqin Zhang, Zerui Wang, Junho Lee, Fei Xu

**Affiliations:** Department of Business Administration, Hoseo University, Asan, Republic of Korea

**Keywords:** guanxi HRM practices, job meaningfulness, turnover intention, voice behavior, ethical leadership

## Abstract

**Introduction:**

Currently, few studies have examined the negative effects of human resource management (HRM) practices on employees’ voice behavior and turnover intention. This study aims to explore the underlying mechanisms and boundary conditions by which guanxi HRM practices influence employees’ turnover intention and voice behavior in the Chinese context, drawing on the conservation of resources theory.

**Methods:**

Using a three-wave survey of 243 employees, we analyzed a moderated mediation model.

**Results:**

The results revealed that guanxi HRM practices were positively associated with turnover intention and negatively associated with voice behavior, with job meaningfulness serving as a mediator in these relationships. Furthermore, we found that ethical leadership mitigated the negative relationship between guanxi HRM practices and job meaningfulness, as well as the indirect effects of guanxi HRM practices on turnover intention and voice behavior through job meaningfulness.

**Discussion:**

These findings deepen our understanding of the interaction between guanxi HRM practices and ethical leadership, as well as their combined effects on job meaningfulness, turnover intention, and voice behavior, and provide practical implications for organizations and managers.

## Introduction

How to promote employees’ voice behavior and reduce their intention to leave remains a central concern for organizations and managers. In today’s VUCA (volatility, uncertainty, complexity, ambiguity) era, intensified environmental uncertainty and competition have made organizations increasingly dependent on employees’ input and proactive suggestions (Raza et al., 2021; Shet, 2024; Xia et al., 2020). Voice behavior, as a form of constructive expression aimed at improving organizational functioning, is vital for preventing crises and enhancing adaptability (Duan et al., 2014). Simultaneously, employees’ intention to leave is a strong predictor of actual turnover behavior (Wang et al., 2020), which can lead to substantial organizational losses. Thus, exploring how to encourage voice behavior and reduce turnover intention is both theoretically significant and practically urgent (Liang et al., 2012).

Previous research has found that positive psychological factors originating from the work environment, such as employee-oriented human resource management and high-commitment human resource management, are important antecedents that promote increased voice behavior and reduced turnover intention among employees (Hu and Jiang, 2018; Yousaf et al., 2018). HRM, as an institutional system guiding critical decisions like promotion and compensation, exerts considerable influence on employees’ perceptions and behaviors (Peccei and Van De Voorde, 2019). However, most existing studies have predominantly focused on the beneficial aspects of HRM, overlooking potential negative impacts that certain HRM practices may have on employee outcomes.

One such practice is guanxi-based human resource management, which has been shown to adversely affect employees (Han and Altman, 2009). Guanxi HRM refers to the extent to which personal relationships, rather than formal rules, influence organizational decisions such as promotions or bonus allocations (Chen et al., 2004). Rooted in traditional Chinese culture, guanxi remains a pervasive phenomenon in the workplace (Chen and Tjosvold, 2006; Hackley and Dong, 2001; Ruan, 2017). Although guanxi can sometimes promote organizational commitment and relational harmony (Liu et al., 2013; Wang et al., 2019), its use in managerial decision-making may also result in perceived procedural injustice (Chen et al., 2011), decreased well-being (Liu and Jia, 2021), and suppressed innovation (Yang et al., 2021). As organizations strive to retain talent and promote employee voice in increasingly dynamic environments (Raza et al., 2021; Xia et al., 2020), it becomes crucial not only to advance positive human resource management practices but also to prevent detrimental practices such as guanxi HRM from undermining these goals (Han and Altman, 2009).

Despite emerging recognition of guanxi HRM practices’ negative consequences, the mechanisms and conditions under which these effects unfold remain underexplored. As a form of unfair HRM practice, guanxi HRM may drain employees’ physical and psychological resources (Yang and Yang, 2020; Yang et al., 2021). Drawing on conservation of resources (COR) theory (Hobfoll, 1998), we argue that employees’ perceived job meaningfulness, as a key psychological resource, may mediate the relationship between guanxi HRM and their work outcomes (Hackman and Oldham, 1976). When employees perceive HRM practices as unfair, their sense of purpose and significance at work may erode, leading to lower engagement and reduced motivation to contribute.

Furthermore, although prior research has called for more investigation into the boundary conditions of guanxi HRM practices’ influence (Chen et al., 2013), few studies have examined how leadership styles may buffer or exacerbate its effects. We address this gap by introducing ethical leadership as a contextual moderator. Ethical leadership, characterized by fairness, consideration of others’ needs and interests, and just managerial practices, differs from broader leadership styles such as transformational or servant leadership. Unlike these approaches, ethical leadership directly addresses the moral and justice-related concerns triggered by guanxi HRM practices, making it especially critical in environments marked by perceived unfairness or uncertainty (Brown et al., 2005; Kalshoven et al., 2013). By reinforcing ethical conduct and promoting fairness in decision-making, ethical leadership may buffer the negative consequences of guanxi HRM, helping employees restore a sense of purpose and voice.

**Figure 1:**
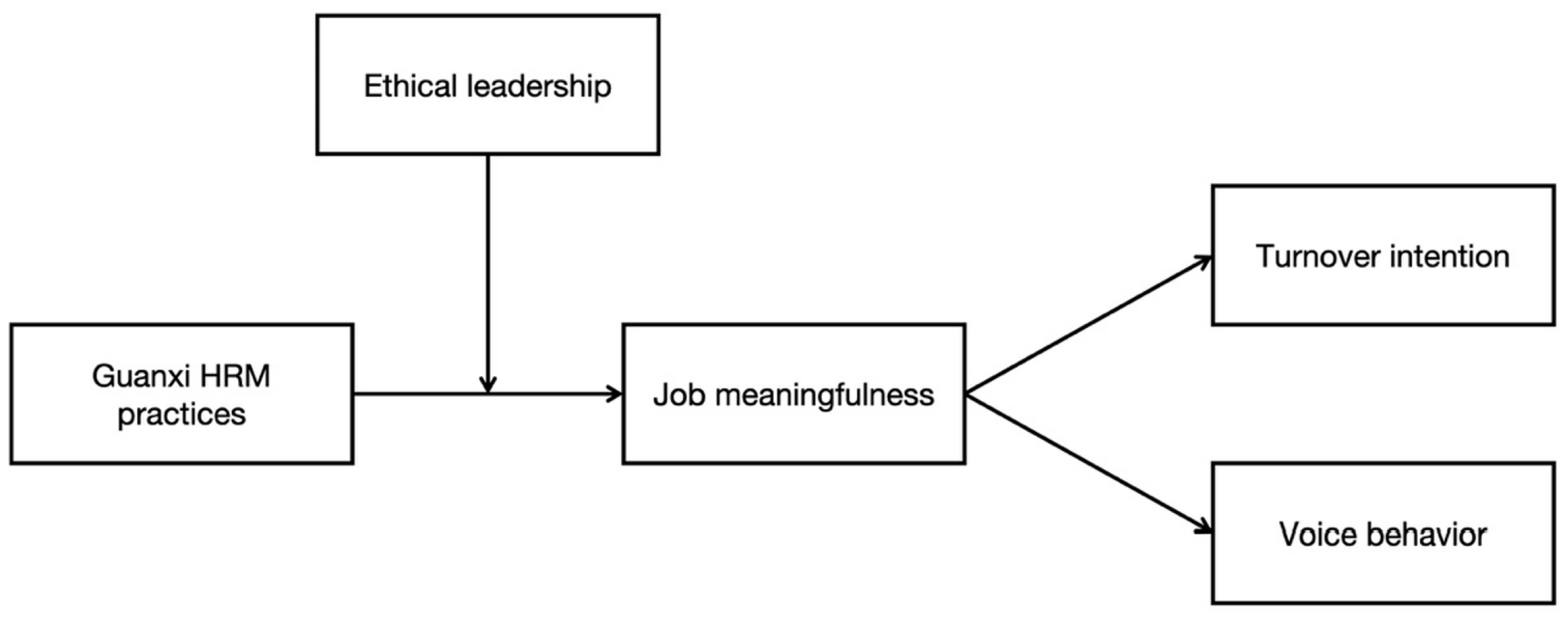
The theoretical model of the study.

This study makes several primary contributions. First, although many studies have examined the outcomes of guanxi HRM, none have simultaneously addressed its relationship with two key employee outcomes: turnover intention and voice behavior. Our study fills this empirical gap. Second, while previous research has shown that diminished job meaningfulness can lead to higher turnover and lower proactive behavior (Leunissen et al., 2018; Soane et al., 2013), the mediating role of job meaningfulness in the guanxi HRM–outcome relationship remains unexplored. Third, we respond to Chen et al.’s (2013) call by examining how ethical leadership moderates the impact of guanxi HRM on employee job meaningfulness. Our integrated model is illustrated in Figure 1.

### Literature review and hypotheses development

#### Guanxi HRM practices, turnover intention and voice behavior

Turnover intention refers to an employee’s conscious and deliberate plan to leave the organization in the near future and is a key predictor of actual turnover (Houshmand et al., 2012; Wang et al., 2020). In contexts dominated by guanxi HRM practices, rewards, compensation, performance evaluations, and promotions are often allocated based on personal relationships rather than objective performance criteria. Employees excluded from these networks tend to perceive procedural unfairness (Chen et al., 2011). Although guanxi is prevalent in Chinese organizations, its ethical legitimacy remains contested, especially when it disadvantages those outside the network (Han and Altman, 2009; Nolan and Rowley, 2020). Meta-analytic evidence shows that perceived procedural injustice increases turnover intention (Cohen-Charash and Spector, 2001; Cropanzano et al., 2001), and unethical HR practices are generally linked to higher turnover rates (Yildiz, 2018). According to COR theory, high stress and exploitative work environments deplete employees’ psychological resources, prompting them to reduce their work engagement or consider leaving to protect their remaining resources (Koenig et al., 2010; Raza et al., 2021).

Voice behavior refers to employees’ voluntary and proactive expression of suggestions, concerns, or opinions aimed at improving organizational functioning (Guzman and Espejo, 2019; Morrison et al., 2011; Van Dyne et al., 2008; Van Dyne and Lepine, 1998). It goes beyond formal job requirements and involves not only proposing ideas but also persisting despite potential resistance (Lam et al., 2022; Ng and Feldman, 2012). This behavior demands substantial psychological resources (Grant and Ashford, 2008; Lam et al., 2022).

In guanxi-dominated environments, employees often experience uncertainty and perceived unfair exploitation, which drains their psychological resources. Consistent with COR theory, when resources are limited, employees tend to reduce discretionary behaviors such as voice to conserve their limited resources. Based on the above analysis, we propose the following hypotheses:

*H1a*. Guanxi HRM practices positively affect employees’ turnover intention.

*H1b*. Guanxi HRM practices negatively affect employees’ voice behavior.

#### The mediating role of job meaningfulness

Hackman and Oldham (1976) defines job meaningfulness as the extent to which individuals perceive their work as inherently valuable and worthwhile. Rather than being an objective construct, meaningfulness is a subjective experience shaped by employees’ perceptions (Steger et al., 2012). As a basic psychological need (Yeoman, 2014), meaningful work supports positive psychological resources such as intrinsic motivation and vitality (Sun et al., 2024), thereby facilitating greater task engagement. Accordingly, fostering a sense of meaningfulness has become a crucial concern for organizations aiming to sustain employee well-being and performance.

However, guanxi HRM practices may undermine work meaningfulness, particularly for employees excluded from informal guanxi networks. When access to key resources (e.g., development opportunities, promotions, or fair recognition) is contingent on personal ties, affected employees may perceive inequity and marginalization. Prior research suggests that a lack of fair treatment and autonomy diminishes employees’ sense of meaning in their work (Wang et al., 2024). Moreover, meaningfulness is a well-documented antecedent of proactive behaviors (Soane et al., 2013) and an important lever in reducing turnover (Trends, 2017; Zhang G. et al., 2023; Zhang Y. et al., 2023).

From the perspective of COR theory, guanxi-based HRM practices introduce psychological strain by eroding employees’ access to valued resources, particularly the psychological resource of meaningfulness. When employees experience diminished work meaning, they may respond by withdrawing effort, reducing discretionary behaviors such as voice, or considering organizational exit as a means of conserving their remaining resources. Based on the above viewpoints, we put forward the following hypotheses:

*H2a*. Job meaningfulness mediates the positive correlation between guanxi HRM practices and employees’ turnover intention.

*H2b*. Job meaningfulness mediates the negative correlation between guanxi HRM practices and voice behavior.

#### The moderating effect of ethical leadership

According to COR theory, employees facing resource depletion must invest resources to prevent further loss and facilitate recovery (Hobfoll, 1989). Ethical leadership represents a valuable resource investment that can help employees cope with such depletion (Tourigny et al., 2019). Brown et al. (2005) defined ethical leadership as demonstrating normatively appropriate conduct through personal actions and interpersonal relationships, while promoting similar behavior among followers via communication, reinforcement, and decision-making. Treviño et al. (2000) emphasize that ethical leaders embody personal virtues such as honesty, integrity, trustworthiness, and fairness, and also practice ethical management, including fair decision-making and balanced reward systems (Brown and Trevino, 2006; Dimitriou and Schwepker, 2019). By fostering fairness and predictability, ethical leadership reduces uncertainty, enhances employees’ sense of control, and promotes belongingness (Rasheed et al., 2024).

In contrast, guanxi-based HRM practices prioritize personal relationships over formal rules and individual competence (Yang et al., 2018; Yang et al., 2021; Zhang et al., 2015). Ethical leadership, by promoting fairness and moral decision-making, directly counteracts the favoritism inherent in guanxi HRM practices (Brown and Trevino, 2006; Cheng et al., 2019). Drawing on COR theory, we argue that ethical leadership can buffer the negative psychological consequences of guanxi HRM, particularly the depletion of employees’ psychological resources such as perceived job meaningfulness. When ethical leadership is high, this buffering effect is expected to be more pronounced. Thus, we hypothesize:

*H3*. Ethical leadership moderates the negative correlation between guanxi HRM practices and job meaningfulness. The higher the ethical leadership, the weaker the negative relationship between guanxi HRM practices and job meaningfulness.

#### The moderated mediation effect of job meaningfulness and ethical leadership

So far, we have discussed how guanxi HRM practices can lead to increased employee turnover intention and reduced voice behavior by undermining employees’ perceptions of job meaningfulness. Furthermore, we have hypothesized that ethical leadership moderates the relationship between guanxi HRM practices and job meaningfulness. Building on these hypotheses, this study further proposes that the indirect effects of guanxi HRM practices on turnover intention and voice behavior through job meaningfulness are moderated by ethical leadership. Specifically, we expect that the strength of the mediated relationship between guanxi HRM practices and employee outcomes varies depending on the level of ethical leadership perceived by employees. Accordingly, we propose the following hypothesis:

*H4a*. The indirect effect of guanxi HRM practices on employee turnover intention via job meaningfulness is moderated by ethical leadership, such that this positive indirect effect becomes weaker when ethical leadership is high.

*H4b*. The indirect effect of guanxi HRM practices on employee voice behavior via job meaningfulness is moderated by ethical leadership, such that this negative indirect effect becomes weaker when ethical leadership is high.

## Methods

### Participants and procedure

The target population of this study consisted of full-time employees from various industries across China. We adopted a convenience sampling strategy and recruited participants through the wenjuanxing platform,^1^ a widely utilized online data collection platform in organizational behavior research (Bai et al., 2024; Wu et al., 2020). The sampling frame consisted of registered users on the platform who met the inclusion criteria, namely being full-time employees and willing to participate in a three-wave longitudinal survey.

To reduce common method bias, we collected data at three time points, with one-week intervals between each wave. Prior to participation, a recruitment announcement briefly explained the purpose and procedures of the study. Participants were assured that their responses would be used exclusively for academic research, that participation was voluntary and anonymous, and that they could withdraw at any time. Completion of the survey was considered as informed consent. The announcement also included the WeChat contact information of a research assistant to address any participant inquiries. Respondents who completed all three waves of the survey received a monetary reward of RMB 4.

At Time 1 (T1), we distributed questionnaires to 500 participants, asking them to report demographic information, perceived guanxi HRM practices, and ethical leadership. A total of 345 valid responses were collected. At Time 2 (T2), these 345 participants were invited to complete a second questionnaire assessing job meaningfulness, resulting in 314 valid responses. At Time 3 (T3), the 314 respondents were asked to report their turnover intention and voice behavior. After data collection, we matched participants’ responses across the three waves using the unique identification codes automatically assigned by the wenjuanxing platform. After excluding invalid responses with excessively short completion times or contradictory information, we retained a final sample of 243 matched and valid cases.

Among the 243 participants, 108 were male (44.4%) and 135 were female (55.6%). In terms of age, 1.8% were under 25 years old, 51.9% were aged 25–29, 23.9% were 30–34, 2.1% were 35–39, and 0.4% were over 40. Regarding educational attainment, 79% held a bachelor’s degree, 1.6% had completed high school or technical secondary school, 14.8% held an associate degree, 3.7% held a master’s degree, and 0.8% held a doctoral degree. Concerning organizational tenure, 53.1% had worked for less than 3 years, 37.4% for 3–6 years, 7.0% for 6–9 years, 2.1% for 9–12 years, and 0.4% for more than 12 years.

### Measures

All items in the current study were originally developed in English and subsequently translated into Chinese using the translation-back translation process established by Brislin (1986). All measures were evaluated on a 5-point scale, from 1 (strongly disagree) to 5 (strongly agree), unless specified otherwise.

#### Guanxi HRM practices

To evaluate guanxi HRM practices, we applied a five-item scale developed by Chen et al. (2004). A sample items was “Many people joined my company through guanxi.” The Cronbach’s alpha for this scale was 0.89.

#### Job meaningfulness

We utilized a three-item scale from Spreitzer (1995) to evaluate job meaningfulness. A sample item includes “The work I do is very important to me.” The Cronbach’s alpha for this scale was 0.87.

#### Turnover intention

To assess turnover intention, we used a four-item scale developed by Chen and Wang (2019). A sample item includes “I often want to leave my present organization or industry.” The Cronbach’s alpha for this scale was 0.91.

#### Voice behavior

We employed 10 items created by Liang et al. (2012) to evaluate voice behavior. A sample item includes “I proactively develop and make suggestions for issues that may influence the unit.” The Cronbach’s alpha for this scale was 0.94.

#### Ethical leadership

We evaluated ethical leadership using the ten-item scale in Brown et al. (2005). A sample item includes “My supervisor listens to what employees have to say.” The Cronbach’s alpha for this scale was 0.92.

#### Control variables

We accounted for gender, education levels, age, and tenure in the current company of these participants, as these characteristics may be related to our key variable, meaningfulness at work (Zhang G. et al., 2023; Zhang Y. et al., 2023).

## Results

### Descriptive statistics and confirmatory factor analysis

Table 1 displays the descriptive statistics, correlation matrix, and internal consistency of all the study variables.

**Table 1:** Descriptive statistics.

Variable	M	SD	1	2	3	4	5	6	7	8	9
1. Gender	1.560	0.498	1								
2. Age	27.2	3.479	0.082	1							
3. Tenure with current company	3.755	2.205	0.067	0.555**	1						
4. Education	2.870	0.518	0.084	0.044	−0.007	1					
5. Guanxi HRM practices	2.752	1.084	−0.183**	−0.182**	−0.127*	−0.158*	1				
6. Job meaningfulness	4.037	0.922	−0.117	0.104	0.070	0.013	−0.199**	1			
7. Turnover intention	2.151	1.086	0.083	−0.197**	−0.231**	0.036	0.440**	−0.390**	1		
8. Voice behavior	3.850	0.885	−0.142*	0.199**	0.157*	−0.048	−0.412**	0.371**	−0.547**	1	
9. Ethical leadership	3.884	0.754	−0.142*	0.005	0.005	0.070	−0.095	0.241**	−0.195**	0.153*	1

*p < 0.05, **p < 0.01.

Additionally, to effectively illustrate that the five proposed constructs in this study are conceptually distinct, we performed a series of confirmatory factor analyses (CFA) using Mplus 8.3 to assess the convergent and discriminant validity of our study variables prior to hypothesis testing. The analytical outcomes reveal that the theoretical five-factor model exhibited the optimal data fit ( χ ^2^ = 795.022, df = 454, χ ^2^/df = 1.751, CFI = 0.934; TLI = 0.928; RMSEA = 0.056; SRMR = 0.044), signifying that the discriminant validity among variables in this study met the requirements.

### Common method bias analysis

To assess the potential impact of common method bias, we conducted a latent method factor analysis by constructing a six-factor measurement model that included a latent common method factor (LCMF), and compared it with the original five-factor measurement model. The results showed that the six-factor model, which included the LCMF, yielded very similar fit indices ( χ ^2^ = 794.867, df = 454, χ ^2^/df = 1.755, CFI = 0.934; TLI = 0.927; RMSEA = 0.056; SRMR = 0.044), with no substantial change observed. These results suggest that common method bias is unlikely to be a serious concern in this study, and the data are deemed appropriate for subsequent analyses.

### Analysis strategy

This study employed the SPSS 27 PROCESS macro to evaluate all hypotheses, adhering to the procedures established by Hayes (2012). We used hierarchical multiple regression analysis to test Hypothesis 1a and Hypothesis 1b. We then constructed a simple mediation model and used PROCESS Model 4 to test Hypothesis 2a and Hypothesis 2b. Finally, we used PROCESS Model 7 to test Hypothesis 3 (moderating effect), Hypothesis 4a, and Hypothesis 4b (moderated mediation effect).

### Hypothesis testing

We performed hierarchical multiple regression analysis to evaluate our hypothesis. Hypotheses 1a posits a positive correlation between guanxi HRM practices and employee turnover intention, while hypothesis 1b claims a negative correlation between guanxi HRM practices and voice behavior. Models 6 and 9 in Table 2 indicate that guanxi HRM practices exhibit a positive and significant correlation with employee turnover intention (*B* = 0.460, *p* < 0.001), whereas they show a negative ad significant correlation with voice behavior (*B* = −0.361, *p* < 0.001). Therefore, hypotheses 1a and 1b are supported.

**Table 2:** Results of hypothesis testing.

Variables	Job meaningfulness				Turnover intention			Voice behavior		
	M1	M2	M3	M4	M5	M6	M7	M8	M9	M10
Gender	−0.238*	−0.301*	−0.236*	−0.227	0.220	0.378**	0.277*	−0.281*	−0.405***	−0.329**
Age	0.243	0.169	0.174	0.167	−0.307	−0.123	−0.066	0.403*	0.258	0.216
Tenure with current company	0.028	0.018	0.016	0.020	−0.258**	−0.232*	−0.226*	0.085	0.064	0.059
Education level	0.034	−0.019	−0.043	−0.019	0.066	0.199	0.193	−0.072	−0.177	−0.172
Guanxi HRM practices		−0.184**	−0.163**	−0.143*		0.460***	0.398***		−0.361***	−0.316***
Job meaningfulness							−0.334***			0.250***
Ethical leadership			0.252**	0.282***						
Guanxi HRM practices × Ethical leadership				−0.140*						
R^2^	0.027	0.070	0.111	0.126	0.072	0.265	0.340	0.070	0.250	0.313

*p < 0.05, **p < 0.01, ***p < 0.001.

Hypothesis 2a and 2b posited that employees’ perceived job meaningfulness mediates the association between guanxi HRM practices and employees’ turnover intention and voice behavior. Table 3 illustrates that, through a bootstrapping procedure involving 5,000 replications, guanxi HRM practices exerted a significant positive indirect effect on employees’ turnover intention via job meaningfulness (indirect effect = 0.061), with a 95% confidence interval (CI) of [0.021, 0.117]. Additionally, guanxi HRM practices demonstrated a significant negative indirect effect on employees’ voice behavior through job meaningfulness (indirect effect = −0.056), with a 95% confidence interval (CI) of [−0.110, −0.018]. The direct influence of guanxi HRM practices on employees’ turnover intention was significant (direct effect = 0.399), with a 95% confidence interval (CI) of [0.287, 0.510], suggesting that job meaningfulness partially mediates the association between guanxi HRM practices and employees’ turnover intention. The direct influence of guanxi HRM practices on employees’ voice behavior was also significant (direct effect = −0.316), with a 95% confidence interval (CI) of [−0.408, −0.223], suggesting that job meaningfulness also partially mediates the correlation between guanxi HRM practices and employees’ voice behavior. Therefore, hypotheses 2a and 2b are supported.

**Table 3:** Results of mediation and moderated mediation effect analyses.

Paths and effects	Estimates	95% confidence intervals	
		LLCI	ULCI
Mediating effect			
GHRMP→JM → TI	0.061	0.021	0.119
GHRMP→JM → VB	−0.046	−0.092	−0.014
GHRMP→JM→TI			
Moderated mediation effect	0.047	0.001	0.104
High EL(+1SD)	0.083	0.034	0.148
Low EL(-1SD)	0.013	−0.050	0.081
GHRMP→JM→VB			
Moderated mediation effect	−0.035	−0.077	−0.001
High EL(+1SD)	−0.062	−0.114	−0.023
Low EL(+1SD)	−0.010	−0.065	0.036

**Figure 2:**
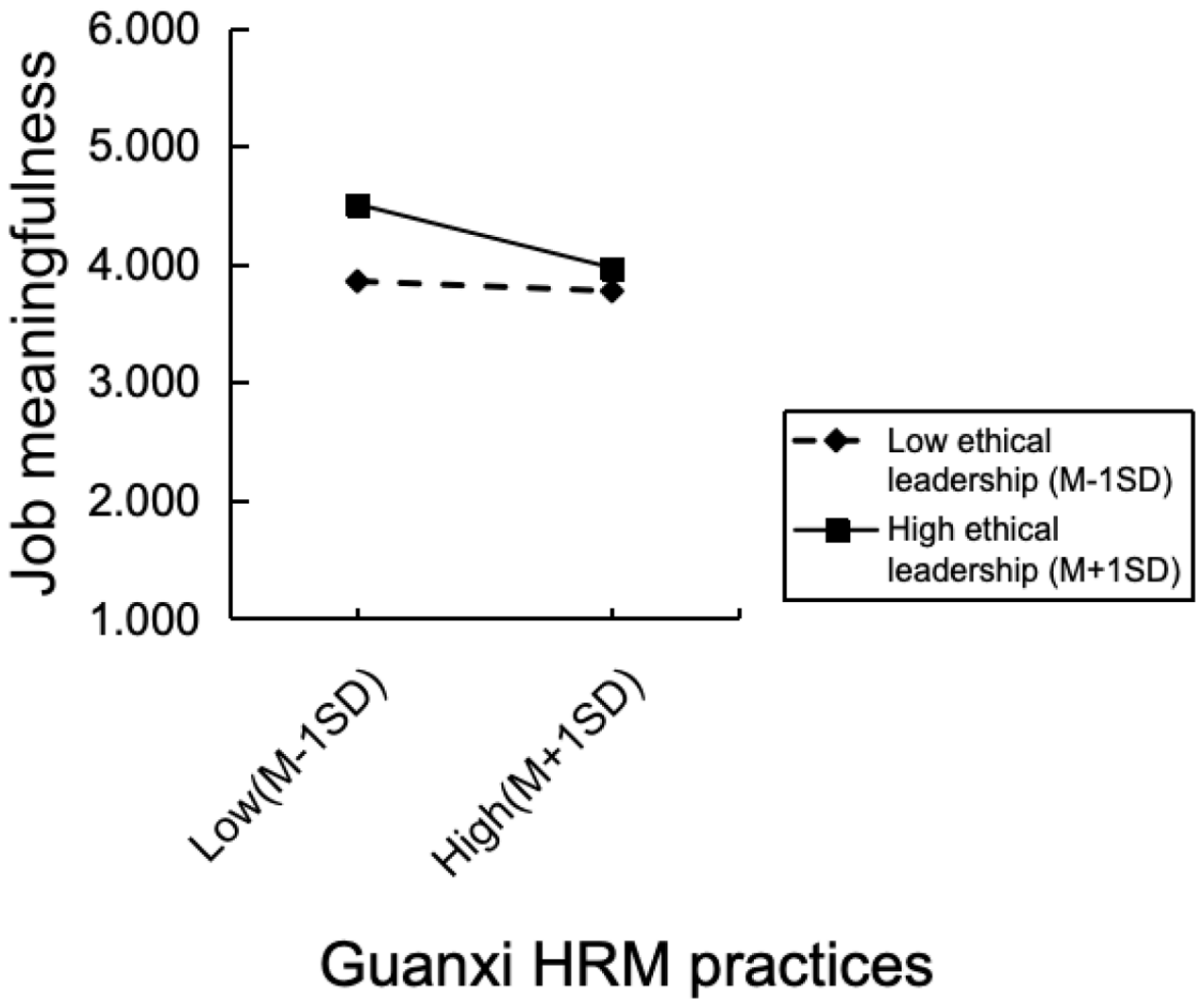
Ethical leadership as a moderator of guanxi HRM practices and job meaningfulness.

Hypothesis 3 posited that employees’ perceptions of ethical leadership moderate the relationship between guanxi HRM practices and job meaningfulness, indicating that this relationship would be diminished for a high level of perceived ethical leadership. Model 4 of Table 2 indicates that the interaction impact of ethical leadership and guanxi HRM practices is negatively correlated with job meaningfulness (*B* = −0.140, *p* < 0.05). We employed Aiken's (1991) methodology to plot this moderation effect in Figure 2. The results reveal that the association between guanxi HRM practices and job meaningfulness diminished when ethical leadership was perceived as high (*B* = −0.248, *p* < 0.001). Moreover, the slope of the regression line connecting guanxi HRM practices to job meaningfulness under conditions of low ethical leadership did not substantially vary from zero (*B* = −0.038, *p* > 0.05). Hypothesis 3 was thus supported.

Hypotheses 4a and 4b posited that ethical leadership moderates the indirect influence of guanxi HRM practices on employees’ turnover intention and voice behavior via job meaningfulness. Specially, the positive indirect effect of guanxi HRM practices on employee turnover intention through job meaningfulness is diminished for employees with higher perceived ethical leadership compared to those with lower perceptions, while the negative indirect effect of guanxi HRM practices on voice behavior through job meaningfulness is also less pronounced for employees with higher perceived ethical leadership than for those with lower perceptions. We employed the PROCESS macro to assess the significance of the conditional indirect effects in testing these hypotheses. Table 3 illustrates that the conditional indirect effects of guanxi HRM practices on turnover intention (indirect effect = 0.083, 95% CI [0.034, 0.148]) via job meaningfulness were positive and significant under high ethical leadership, whereas the conditional indirect effects of guanxi HRM practices on voice behavior (indirect effect = −0.062, 95% CI [−0.114, −0.023]) through job meaningfulness were negative and significant under high ethical leadership. The indirect impacts with low ethical leadership were insignificant for turnover intention (indirect effect = 0.013, 95% CI [−0.050, 0.081]) and voice behavior (indirect effect = −0.010, 95% CI [−0.065, 0.036]). This evidence provides support for Hypotheses 4a and 4b.

## Discussion

This study investigates the impact of guanxi HRM practices on employees’ voice behavior and turnover intention through the lens of conservation of resources theory. Our findings identify guanxi HRM practices as a novel antecedent influencing both turnover intention and voice behavior, with job meaningfulness serving as a critical mediating mechanism. Moreover, the negative effect of guanxi HRM practices on job meaningfulness is attenuated under conditions of high ethical leadership. Based on these results, we discuss the theoretical contributions and practical implications of this research.

### Theoretical implications

This study contributes to the literature on human resource management through several key theoretical extensions. First, we broaden the perspective on the potential negative consequences of HRM practices, particularly their impact on employees’ voice behavior and turnover intention. While prior research has primarily emphasized how HRM fosters positive psychological states and cognitive mechanisms to encourage voice behavior and reduce turnover intention, our findings highlight a less explored dimension: the detrimental effects of HRM practices. In doing so, this study responds to ongoing scholarly calls to uncover the “dark side” of HRM and fosters a more nuanced and comprehensive understanding of its complex nature (Veld and Alfes, 2017; Xu et al., 2020).

Second, we advance the literature on guanxi by addressing its adverse and potentially unethical implications. Although much of the existing research has underscored the benefits of guanxi, an increasing number of scholars have urged further exploration into its negative outcomes (Chen et al., 2004; Yang, 2014; Yang et al., 2018). Drawing on COR theory, our study demonstrates that guanxi-based HRM practices can place significant psychological strain on employees, prompting turnover intention and suppressing voice behavior. This insight enriches current understanding of how guanxi-driven decisions can undermine organizational functioning.

In addition, we respond to the growing emphasis on job meaningfulness, particularly among younger generations of employees who prioritize purpose and social impact in their work (Sun et al., 2024). According to COR theory, job meaningfulness functions as a vital psychological resource. When employees perceive exploitation or unfair treatment stemming from guanxi HRM practices, this resource may become depleted. To protect their remaining resources, employees may reduce voice behavior and increase turnover intention. Thus, our study not only sheds light on the deleterious impact of guanxi HRM practices but also contributes to the literature by positioning job meaningfulness as a critical mediating mechanism, addressing the research agenda proposed by Rosso et al. (2010) and extended by Wu et al. (2024).

Finally, we examine the boundary conditions under which guanxi HRM practices influence employee outcomes. In response to Chen et al. (2013)‘s call for more research on contextual moderators of guanxi HRM, and drawing on COR theory to address scholars’ appeals for expanding potential background factors that may inhibit or facilitate the resource conservation process (Hagger, 2015; Xu et al., 2020), we examine ethical leadership as a key moderator. Our findings reveal that ethical leadership, as a form of resource investment, can buffer the negative effects of guanxi HRM practices by preserving employees’ perceived job meaningfulness. Furthermore, we show that ethical leadership moderates the indirect relationship between guanxi HRM and employee outcomes, including turnover intention and voice behavior. In doing so, this study deepens our understanding of how the mediating role of job meaningfulness varies across different levels of ethical leadership.

### Practical implications

Our findings indicate that guanxi HRM practices lead to increased employee turnover intention and reduced voice behavior. Because both outcomes can negatively affect organizational effectiveness, we propose the following practical implications.

First, organizations and managers should implement measures to curb HR decisions based on guanxi rather than employees’ abilities or performance. For example, unfair resource allocation driven by guanxi can be mitigated by establishing accountability mechanisms that punish managers who engage in such practices.

Second, our results show that guanxi HRM practices diminish employees’ job meaningfulness, which in turn increases turnover intention and reduces voice behavior. Therefore, when guanxi-based management decisions are already present, organizations should promptly intervene by enhancing the transparency of HR processes. Making decisions openly and transparently, clearly specifying who made the decision, when, and how, can reduce employees’ feelings of uncertainty and unpredictability, thereby enhancing their sense of job meaningfulness.

Finally, given that guanxi is deeply embedded in Chinese culture and difficult to eliminate entirely (Ruan, 2017), our study highlights the buffering role of ethical leadership in mitigating its negative effects on employees. Additionally, individuals with lower moral standards tend to rely more on guanxi (Ho and Redfern, 2010). Thus, we recommend that organizations prioritize the selection and promotion of managers and employees with high ethical awareness in HR decisions such as recruitment and promotion. Cultivating an ethical work climate can better restrain the development and influence of personal guanxi within organizations.

### Limitations and future research

Our study has several limitations that future research should address. First, we relied on self-reported data, which may raise concerns about common method variance due to a single data source and social desirability bias (Podsakoff et al., 2012). We mitigated this through anonymous surveys and a time-lagged design, and latent method factor analysis suggested CMV was not a major issue. Nonetheless, future studies should use multi-source data (e.g., supervisor or peer ratings) and experimental designs to further reduce CMV.

Second, we measured job meaningfulness with a brief three-item scale, which may not fully capture its complexity. Future research could adopt more comprehensive, multidimensional measures, such as the Work and Meaning Inventory (Steger et al., 2012), or qualitative methods. Exploring additional mediators could also provide a fuller understanding of how guanxi HRM affects turnover intentions and voice behavior.

Third, while we tested some boundary conditions, other moderators may influence employee responses. Future studies should investigate more moderators to reveal diverse coping strategies under guanxi-based HRM.

In addition, guanxi is culturally specific to China, limiting generalizability. However, similar informal favoritism exists in other contexts with weak formal institutions, such as Japan and the Republic of Korea (Cho and Yoon, 2001; Lebra, 1976), and Chinese state-owned enterprises (Park and Luo, 2001; Xin and Pearce, 1996). Understanding how perceived unfair HRM affects behavior and leadership’s mitigating role has broader relevance. Future research should examine indigenous HRM practices in different countries to better understand how local concepts shape management processes, thereby enhancing the effectiveness of cross-cultural management (Ren et al., 2024).

Finally, COR theory explains how employees cope with resource-draining human resource management (HRM) practices, but Chinese cultural norms are equally important (Hwang, 1987; Farh et al., 1998). In China’s high power distance context, employees often internalize dissatisfaction out of respect for hierarchy, leading to depletion of psychological resources (Farh et al., 2007; Hofstede, 2001; Morrison and Milliken, 2000). Collectivism emphasizes harmony and suppresses conflict, which increases emotional strain (Eckhardt, 2002; Triandis, 2018). The face-saving culture further reduces employees’ willingness to speak up, as they fear embarrassing superiors or damaging their reputation (Hwang, 1987; Dai et al., 2022). These cultural factors may exacerbate the negative effects of guanxi-based HRM, increasing turnover intentions and reducing proactive behaviors. Future research should incorporate power distance, harmony, and face-saving cultural dimensions into the COR framework to better explain how culture influences resource conservation processes in non-Western contexts.

## Data Availability

The original contributions presented in the study are included in the article/Supplementary material, further inquiries can be directed to the corresponding author.

## Footnotes

^1^ https://www.wjx.cn
